# A deep learning approach for keratoconus detection using spatio-temporal features from corneal imaging

**DOI:** 10.1038/s41598-026-56383-y

**Published:** 2026-06-01

**Authors:** Arkadiusz Syta, Tomasz Chorągiewicz, Jakub Gęca, Robert Karpiński, Dominika Wróbel-Dudzińska, Arkadiusz Podkowiński, Marcin Maciejewski, Dariusz Głuchowski, Katarzyna E. Jonak, Robert Rejdak, Kamil Jonak

**Affiliations:** 1 Faculty of Mathematics and Information Technology, Department of Technical Computer Science, Lublin University of Technology, Nadbystrzycka 38, Lublin, 20-618 Poland; 2 Chair and Department of General and Pediatric Ophthalmology, Medical University of Lublin, Chmielna 1, Lublin, 20-079 Poland; 3 Faculty of Electrical Engineering and Computer Science, Department of Electrical Drives and Machines, Lublin University of Technology, Nadbystrzycka 38A, 20-618, Lublin, Poland; 4 Faculty of Mechanical Engineering, Department of Machine Design and Mechatronics, Lublin University of Technology, Nadbystrzycka 36, Lublin, 20-618 Poland; 5 Institute of Medical Sciences, The John Paul II Catholic University of Lublin, Konstantynów 1F, Lublin, 20-708 Poland; 6 Da Vinci NeuroClinic, Tomasza Zana 11A, Lublin, 20-601 Poland; 7 Faculty of Electrical Engineering and Computer Science, Department of Electronics and Information Technology, Lublin University of Technology, Nadbystrzycka 38A, Lublin, 20-618 Poland; 8 Faculty of Mathematics and Information Technology, Department of Applied Informatics, Lublin University of Technology, Nadbystrzycka 38, Lublin, 20- 618 Poland; 9 Doctoral School, The John Paul II Catholic University of Lublin, Al. Racławickie 14, Lublin, 20-950 Poland

**Keywords:** Deep learning, Keratoconus, Cornea, Biomechanics, CNN-RNN, Fuzzy logic, Corneal diseases, Machine learning

## Abstract

Keratoconus is a progressive corneal disease that requires early and accurate detection to prevent severe visual impairment. This study presents a deep learning-based classification model for distinguishing between healthy and keratoconic eyes using dynamic corneal imaging data from the CORVIS system. A hybrid CNN-RNN architecture was developed, combining a fine-tuned InceptionV3 network for spatial feature extraction with a recurrent LSTM module to capture temporal patterns across image sequences. To ensure robust evaluation, a 10-fold stratified cross-validation strategy was employed, with data splits performed at the patient level to avoid data leakage. The model achieved an average accuracy, precision, recall, and F1-score of approximately 0.90 across folds, demonstrating strong generalization performance. Boxplot visualizations of metric distributions further confirmed model stability and revealed minimal performance variance. Class-wise analysis showed high effectiveness in detecting both healthy and keratoconic cases, although slightly greater variability was observed in the classification of healthy eyes. These results indicate that the proposed method is a promising tool for keratoconus screening and may complement existing diagnostic workflows. Further validation on external datasets is recommended prior to clinical deployment.

## Introduction

The cornea is the crucial element of the refractive system of the eye, accounting for its 70% of the total refractive power. The quality of vision depends on its shape and transparency, as well as on the stability and integrity of the corneal structure, which result from the biomechanical properties of the cornea^1,2^. They determine its response to stress and deformation. This process depends on the cornea’s viscoelastic properties. Cornea can be considered as complex biomechanical composite. The corneal stroma, accounting for more than 90% of its thickness, consists of 300–500 lamellar sheets made of unbranched collagen fibrils stretching across the entire cornea from limbus to limbus. Every sheet has a very regular organization: all fibrils are parallel to each other and regularly spaced^3,4^.

Biomechanical properties of the cornea are important for intraocular pressure (IOP) measurement, and play a role in such clinical conditions as keratoconus, corneal refractive surgery, and glaucoma^5^.

Keratoconus is a progressive, non-inflammatory disorder of the cornea characterized by thinning, cone-shaped convexity, and changes in biomechanical parameters^6^. This leads to irregular astigmatism, impaired vision, and decreased visual acuity. The etiology of corneal cone is not yet fully understood, but the influence of genetic, biomechanical, and environmental factors is indicated^7^. Early diagnosis is crucial to halt disease progression and implement appropriate treatment^8,9^.

Traditional diagnostic methods used to date are mainly based on analyzing the shape and thickness of the cornea using topography and tomography (Scheimpflug, OCT)^10^. Although these techniques allow the evaluation of advanced corneal cone, their effectiveness in detecting early changes is limited^8,11,12^. Consequently, methods based on corneal biomechanical properties are becoming increasingly important.

One modern diagnostic tool is the Ocular Response Analyzer (ORA), which measures the cornea’s response to dynamic deformation by determining corneal hysteresis coefficients (CH) and coefficients of resilience (CRF)^13,14^. In the case of the corneal cone, these values are reduced, indicating structural weakness in the tissue^15^.

Another commonly used diagnostic method is Corvis ST (Corneal Visualization Scheimpflug Technology), which records the dynamic deformation of the cornea using an air jet and Scheimpflug imaging^16,17^. This system provides a series of biomechanical indices, including stiffness and deformation indices, that enable earlier detection of degenerative changes in corneal structure.

Corvis ST (Oculus Optikgeräte GmbH, Wetzlar, Germany) is a non-contact semi-automatic device designed to analyze corneal biomechanics in vivo. It uses an air pulse to induce central corneal compression and deformation, and obtains real-time corneal deformation data from a high-speed Scheimpflug camera that records 4300 high-resolution horizontal images per second^18–20^. A slow-motion reconstruction of corneal deformation is displayed on the device.

The Scheimflug image allows for the evaluation of central corneal thickness (CCT). For ocular biomechanical parameters, two corneal applanations are applied. During the first applanation, intraocular pressure (IOP) is calculated^21^. Corneal biomechanical parameters include the first/second applanation time (A-time1/A-time2), the first/second applanation length (A-length1/A-length2), corneal velocity during the first/second applanation moment (Vin/Vout), highest concavity time, highest concavity-radius (HC-radius), deformation amplitude (DA), distance between bending points of the cornea (peak distance - PD), and the concave radius of curvature (R) at the point of highest concavity.

The application of Scheimpflug imaging to identify corneal biomechanical parameters using artificial intelligence methods, along with results from other studies (including multimodal data, i.e., numerical and imaging data), enables effective classification of keratoconus. Extracting the basic geometric features of the cornea based on the changing curves of its deformation during the examination enabled the correct diagnosis of the condition with 99% accuracy using a neural network^22^. Expanding the biomechanical features of the cornea using topographic data (obtained with the Pentacam device) enabled the Random Forest model to achieve high classification accuracy (89%)^23^. A similar combination of feature sets enabled the authors to identify the optimal diagnostic indicator with an AUC of 93%^24^. Comparing data from three devices (Sirius, Pentacam, and Corvis) identified indicators with the highest diagnostic accuracy (AUC = 91% for Sirius and 82% for Corvis)^25^. Transfer learning is also applied in the classification of keratoconus. The authors compared the diagnostic accuracy of the ResNet152, VGG16, and InceptionV3 models on images obtained from the Corvis device, with ResNet152 achieving the highest AUC (99%)^26^. The latest review^27^ of the application of artificial intelligence models in the diagnosis of corneal diseases highlights their high diagnostic accuracy and notes limitations related to the lack of external validation and population heterogeneity. In the context of the visual system, fuzzy logic has been utilized in various applications. Recent studies further support the growing importance of artificial intelligence and wearable technologies in the broader context of keratoconus diagnostics, monitoring, and risk-factor assessment. In particular, machine learning and deep learning approaches have been applied not only to corneal imaging and progression prediction, but also to the objective detection of eye-rubbing behavior, which is clinically relevant for the development and progression of ectatic disease^28–30^. These findings are consistent with the rationale of the present study, which uses dynamic CORVIS ST imaging and a CNN-RNN architecture to extract spatial and temporal features for keratoconus classification. The integration of calibration data and near-infrared (NIR) camera data using fuzzy logic has enhanced the gaze-tracking system regardless of marker type^31^. In a similar application involving the detection of the pupil center and corneal reflection, a fuzzy system that accounted for the driver’s head rotation effectively reduced detection errors under vehicle conditions^32^. Fuzzy reasoning has also found applications in the diagnosis of various ocular conditions. The comparison of three selected classification strategies - using an artificial neural network, a fuzzy classifier, and a neuro-fuzzy classifier - enabled achieving 100% specificity in the diagnosis of cataracts, iritis and cyclitis, and corneal opacities^33^. A system integrating artificial neural networks with adaptive neuro-fuzzy inference systems enabled classification with up to 100% accuracy (in certain classes) in diagnosing abnormalities in corneal layers^34^. This demonstrates that integrating medical imaging with additional diagnostic information via fuzzy systems can enhance diagnostic efficacy, serving as a precursor to the development of automated diagnostic systems. An example of such integration can be found in the work^35^, where the authors differentiated healthy patients from those with diabetic neuropathy using measurements of nerve fiber length. The integration of corneal topography imaging data (using the Pentacam device) with a fuzzy classifier enabled the diagnosis of keratoconus in a multi-class classification process with very high accuracy, reaching up to 98%^36^. The above examples demonstrate that incorporating a fuzzy system and additional diagnostic information can lead to more effective diagnoses. In the context of corneal medical image processing, fuzzy reasoning can also be applied at an earlier stage to remove noise, eliminate artifacts, and sharpen both the anterior and posterior edges of the cornea, which is crucial for further analysis of anatomical structures and biomechanical properties. In^37^, the authors proposed an Adaptive Cascaded Decoder based on dynamic convolution, which enhances medical image segmentation by adaptively adjusting the receptive field, combining local and global image features, and sharpening blurred boundaries and improving contrast.

## Materials and methods

### Study participants

The study using the Corvis ST device involved two groups of patients seen in an outpatient clinic: a pilot group diagnosed with keratoconus (57 eyes) and a control group (47 eyes). The research was conducted at the Department of Ophthalmology, Medical University of Lublin, from March to August 2024.

Before participation, each patient provided written informed consent. The study was conducted in accordance with Good Clinical Practice (GCP) guidelines. It was approved by the Local Ethics Committee of the Medical University of Lublin (approval number KE-0254/98/03/2023) and conducted in compliance with the Declaration of Helsinki.

Preliminary ophthalmic examinations included: objective and subjective refraction, slit-lamp examination, intraocular pressure measurement, and corneal tomography. Patients included in the study met the following inclusion criteria:

1. **Healthy eyes**:

- Normal corneal tomography and topography results (K max < 47 D, lower-upper difference < 1.5 D, oblique axis radial index < 22º).
- Normal elevation maps of the anterior and posterior corneal surfaces.
- Normal corneal thickness distribution (CCT > 480 μm).
- No corneal scarring.
- No clinical signs of keratoconus.
- No family history of keratoconus.

2. **Eyes with keratoconus**:

- Abnormal corneal tomography and topography results (K max > 47 D, lower-upper difference > 1.5 D, oblique axis radial index > 22º).
- Abnormal elevation maps of the anterior and posterior corneal surfaces.
- Central or inferior corneal protrusion.
- Thin cornea.
- No corneal scarring.

Exclusion criteria included various types of corneal ectasias (such as pellucid marginal degeneration (PMD) or keratoglobus), corneal endothelial diseases, previous ocular surgeries, and eye infections.

### Data Acquisition

All examinations were performed at the Department of Ophthalmology, Medical University of Lublin, using the Corvis ST device (Oculus Optikgeräte GmbH, Wetzlar, Germany) according to the manufacturer’s standard protocol. An experienced operator acquired measurements under controlled conditions.

For each eye, the full set of Corvis ST parameters was recorded. In addition, the dynamic corneal deformation sequence induced by the air puff was exported for further analysis. The recordings captured the successive phases of corneal deformation, from the initial state through inward applanation, maximum concavity, outward applanation, and recovery.

All recordings were reviewed for technical quality before inclusion. Only complete examinations with sufficient image quality were included in further analysis. The data were anonymized before processing. Each eye was treated as a separate case, while cross-validation splitting was performed at the patient level to prevent data leakage.

### Video processing

This article presents a study on the classification of videos from the CORVIS device examination for the diagnosis of keratoconus. The input dataset consisted of 104 videos: 57 representing eyes with keratoconus and 47 representing healthy cases. Each video obtained from the CORVIS examination contained 139 frames at 200 × 576 pixels. The video lengths and frame sizes were consistent across all examinations, so no standardization was required.

Proper data preparation is a crucial step in training and implementing artificial intelligence algorithms for medical imaging. Medical images often contain noise or glitches that hinder the algorithm’s ability to learn and recognize patterns in the training data. Preprocessing can improve the classification performance of medical images, as highlighted by the authors in^37^. Furthermore, using appropriate image preprocessing methods can accelerate training and reduce hardware requirements, which is critical in some applications. An example can be found in^38^, where the authors explore the implementation of classification and segmentation models for MRI images on edge devices such as Nvidia Jetson, Raspberry Pi, and an Android smartphone.

Video preprocessing was performed using the OpenCV library^39^. Each video displays essential information about the examination, including patient data, the examined eye, and the test date. Additionally, each frame contains the manufacturer’s watermark (Oculus). Therefore, removing these text elements from the videos becomes essential, as the leakage of personal patient information or the examined eye could lead the algorithm to treat this data as relevant for classification, which is unacceptable. Moreover, the CORVIS device manufacturer’s logo (watermark) is a bright, sharp-edged object, which could prevent the algorithm from detecting subtle corneal edges and their deformation over time, crucial features for diagnosis.

Therefore, text and logo removal were carried out using the following procedure:

1. Edge detection using the Canny algorithm.
2. Enhancement of thin text edges using dilation.
3. Contour detection.
4. For each detected contour, a bounding rectangle is computed, and conditions related to the minimum and maximum letter sizes and aspect ratio (width-to-height ratio) are checked.
5. Based on the elements from step 4, a mask is created, and text is removed using inpainting, i.e., filling in the masked regions based on their surroundings (background appearance estimation).

In the first stage, the Canny edge detector is applied to identify object boundaries with high contrast, which are characteristic of textual elements. Subsequently, morphological dilation is performed on the edge image to enhance thin and discontinuous letter structures, thereby improving the coherence of their representation. In the processed image, external contours are detected, and a bounding rectangle is computed for each. Candidate text regions are then filtered based on geometric features such as width, height, and the aspect ratio. Contours that meet the predefined criteria are projected onto a binary mask, defining regions to be removed. In the final step, an image inpainting algorithm is applied to reconstruct the marked regions using information from their immediate surroundings. As a result, a frame with removed textual elements and a restored background is obtained.

**Fig. 1:**
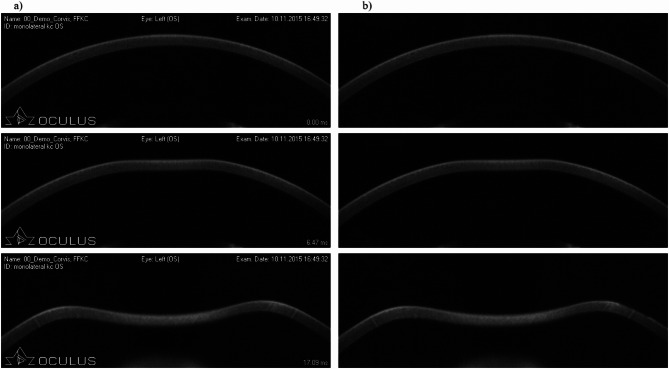
Representative CORVIS ST video frames before (a) and after (b) preprocessing, illustrating effective removal of textual overlays and device watermark while preserving diagnostically relevant corneal geometry and deformation patterns.

The results of the operations can be observed in the example video frames (Fig. 1).

A comparison of sample images before preprocessing (Fig. 1a) and after its application (Fig. 1b) clearly demonstrates the effectiveness of the implemented processing algorithms. All textual annotations have been successfully removed, ensuring full anonymization of each image while preserving the characteristic corneal curvature observed at different stages of the examination.

**Fig. 2:**
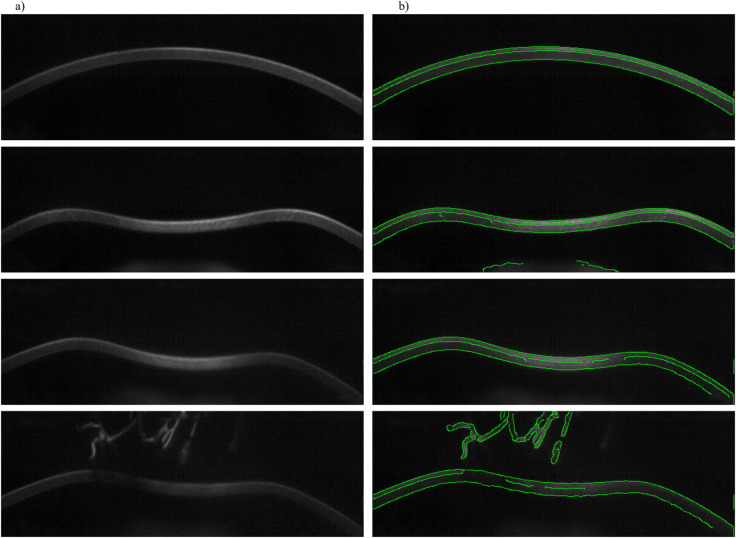
Examples of corneal edge detection results using the Canny-based preprocessing pipeline: (a) raw frames and (b) corresponding edge-enhanced images highlighting anterior and posterior corneal boundaries, along with visible artifacts such as reflections and blinking-induced noise.

In the diagnosis of keratoconus, some of the most important parameters are the shape, thickness, and biomechanics of the cornea. Therefore, the corneal edges are a crucial diagnostic feature for the algorithm, as they enable assessment of both corneal thickness and the degree of deformation. The CORVIS device overlays special curves on images taken during the examination, fitting them to the corneal edges; however, these overlays are not applied to the video frames generated by the device. For this reason, the Canny edge detection algorithm^40^ was applied, along with a corresponding filter applied to each video frame, to facilitate the extraction of these features from the analyzed image. An example of applied filters is shown in Fig. 2.

Unfortunately, CORVIS examination videos may contain various distortions and glitches - for instance, the patient may blink or move during the test, which further causes image blurring. Therefore, selecting appropriate parameters for the Canny algorithm is a compromise: to detect corneal edges properly, the algorithm may also detect glitches as edges in some recordings. As a result, in certain cases, additional disturbances - or even parts of the patient’s eyelashes (when blinking during the exam) - are also detected as edges.

The example presented in Fig. 2 illustrates the next stage of image processing, involving edge enhancement and corneal contour extraction, and reveals varying outcomes. In the first case (Fig. 2b, top) - corresponding to the initial phase of the examination - both the anterior and posterior corneal surfaces are clearly delineated. However, some additional noise is present due to light reflections. In the second case (Fig. 2b, middle) - a subsequent phase of the examination where corneal deformation is already visible - the extracted contour is also correctly marked. In the final case (Fig. 2b, bottom), where corneal deformation is again present, the curvature is properly extracted; however, artifacts resulting from blinking are also visible.

The final step in the preprocessing of individual video frames was resizing the images to 200 × 200 pixels, a size more suitable for most algorithms and that also enabled faster training due to the reduced number of pixels. Next, every video frame was also normalized.

The next stage involved processing the entire videos. While the preprocessing of individual frames aimed to facilitate the extraction of spatial features such as textures, edges, shapes, and spatial arrangements, the classification of an entire video also depends on temporal features –the variability of frames over time, such as object motion, changes in brightness or structure, and inter-frame correlations. It was observed that among the 139 frames recorded during the examination, only some contain useful temporal information related to corneal biomechanics. The initial frames depict a static state in which the patient is waiting for the examination to begin, and the cornea remains still (except for minimal movement, for example, from breathing). Then, an air puff is applied to deform the cornea, and the following frames capture the sequence from the initial state through maximum deformation back to the initial state.

In addition to the dynamic video sequence, the Corvis ST device provides images corresponding to three key corneal states: the initial state, applanation, and highest concavity. Together with the intermediate biomechanical response, these phases contain the most diagnostically relevant information. Each examination video consisted of 139 frames. The initial part of the sequence corresponded to the baseline state before the air puff, followed by the deformation phase leading to maximum concavity, and finally the recovery phase, during which the cornea returned toward its original shape. Because the initial and final parts of the sequence contained highly similar spatial information with limited biomechanical variation, including all frames during feature-extractor training could introduce redundancy and bias the model toward repetitive patterns. Therefore, to reduce memory requirements and training time while preserving the most dynamically informative part of the sequence, only the 55-frame segment corresponding to the main corneal deformation phase was used to train the feature-extraction model. In contrast, the sequential model based on a recurrent neural network (RNN) was trained using the complete 139-frame examination sequences. While the reduced 55-frame subset was sufficient for learning spatial representations, the recurrent component operated on the full temporal sequence to preserve the complete temporal structure of the examination, including the baseline, deformation, and recovery phases.

For consistency, the additional baseline models used in the final comparative analysis (3D CNN and Transformer-based architectures) were trained using the same selected 55-frame segment and the same patient-level cross-validation protocol.

## Model Architecture

Due to the complexity of image classification and the relatively small dataset available, a 10-fold stratified cross-validation approach was used. This method involves dividing the data into 10 equal parts, using 9 for training and 1 for validation, and changing the validation subset in each iteration. The stratified variant ensures that each subset maintains approximately the same ratio of diseased to healthy cases, with the splits performed at the patient level rather than at the level of individual image files. This approach allowed the algorithm to avoid bias toward a particular test set and to tune model parameters properly. It is also important to note that video preprocessing was performed before cross-validation. Therefore, all decisions regarding data preparation were made independently of the division into training and validation sets.

Among the available methods that support image classification using both spatial and temporal features, the following are the most popular:

- **CNN-RNN** - a model that combines convolutional neural networks for extracting spatial features with recurrent networks for classifying based on their temporal evolution across video frames.
- **3D CNN** - a convolutional neural network that takes sequences of frames (or entire videos) as input, simultaneously analyzing spatial features and temporal context^41^.
- **Transformer** - a special model using a self-attention mechanism, allowing each input element (e.g., an image patch) to reference other elements and determine which ones are most relevant^42^.

This study adopted the first approach - a CNN-RNN model - while also leveraging transfer learning. The CNN-RNN architecture was selected as a compromise between representational capacity and model complexity. Compared with 3D CNN architectures, which jointly process spatial and temporal information using computationally expensive volumetric convolutions, the proposed approach separates spatial feature extraction from temporal sequence modeling, thereby reducing the number of trainable parameters and the risk of overfitting. Compared with Transformer-based architectures, the CNN-RNN model requires substantially fewer training samples and exhibits more stable learning behavior under limited-data conditions. This is particularly important in the present study, given the relatively small patient-level dataset available for training and validation.

For spatial feature extraction, the pre-trained InceptionV3 model^43^, trained on the ImageNet dataset, was used. Due to the specific nature of images generated during CORVIS examinations, the last 20 layers of the model were additionally fine-tuned on the training set for each fold to better adapt the feature extractor for keratoconus detection. The full model was not retrained to reduce training time and avoid overfitting.

Training of the feature extractor involved adding a densely connected final layer with a single neuron and a sigmoid activation function. The model was trained for 100 epochs using the Binary Crossentropy loss function and the Adam optimizer^44^ with a learning rate of 0.001 and a batch size of 4. Two callbacks were defined during training: the first, EarlyStopping, monitored the validation loss with a patience of 5 epochs; the second, ModelCheckpoint, saved the best model (by validation loss) to a file for use as a feature extractor in the final model.

**Fig. 3:**
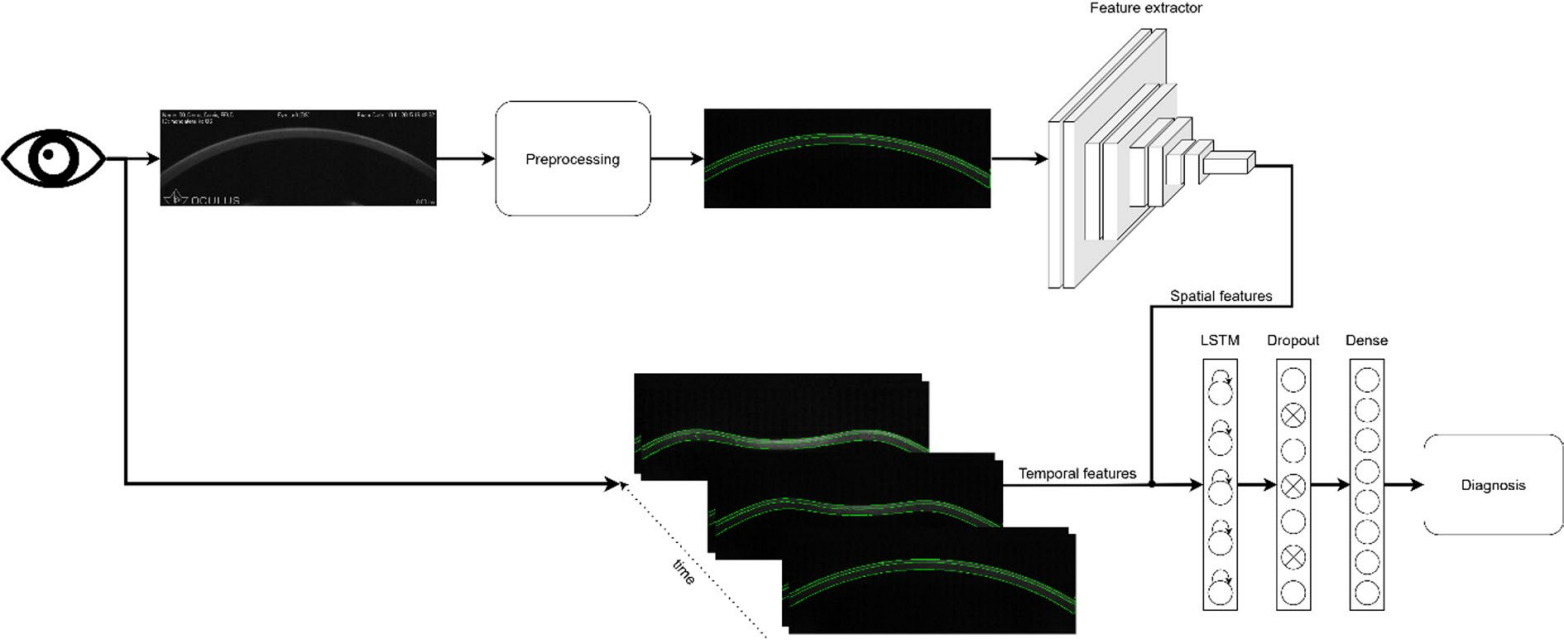
Schematic representation of the proposed CNN-RNN architecture combining spatial feature extraction using a fine-tuned InceptionV3 network with temporal modeling via an LSTM module for classification of corneal deformation sequences.

The final layer responsible for binary classification was removed from the trained model, allowing it to be used for extracting spatial features from individual video frames. However, spatial features alone are not sufficient. Therefore, the article proposes a two-part model: spatial feature extraction using InceptionV3, followed by classification with a recurrent neural network. The architecture of the entire model is shown in Fig. 3. In the sequential part of the model, one LSTM layer with 16 units was used, followed by a 20% dropout layer for regularization, then a densely connected layer with 16 neurons and ReLU activation, and finally a classification layer with 1 neuron and a sigmoid activation function. This compact LSTM configuration was intentionally selected to limit the number of trainable parameters and reduce the risk of overfitting, particularly given the limited patient-level dataset. This recurrent network was trained for 100 epochs using the Binary Crossentropy loss function and the Adam optimizer with a learning rate of 0.0001 and a batch size of 32. A ModelCheckpoint callback was also applied to allow retrieval of the best-performing model based on validation loss. No data augmentation or class-balancing techniques were applied during model training, as it was decided that, for the sake of clinical reliability, the model should operate exclusively on real data from clinical examinations. Model hyperparameters were optimized via an iterative trial-and-error procedure, in which successive configurations were evaluated based on training dynamics (e.g., convergence rate and stability) and validation performance. This empirical approach enabled incremental adjustments to parameter values, facilitating the identification of settings that improved generalization.

## Results

**Fig. 4:**
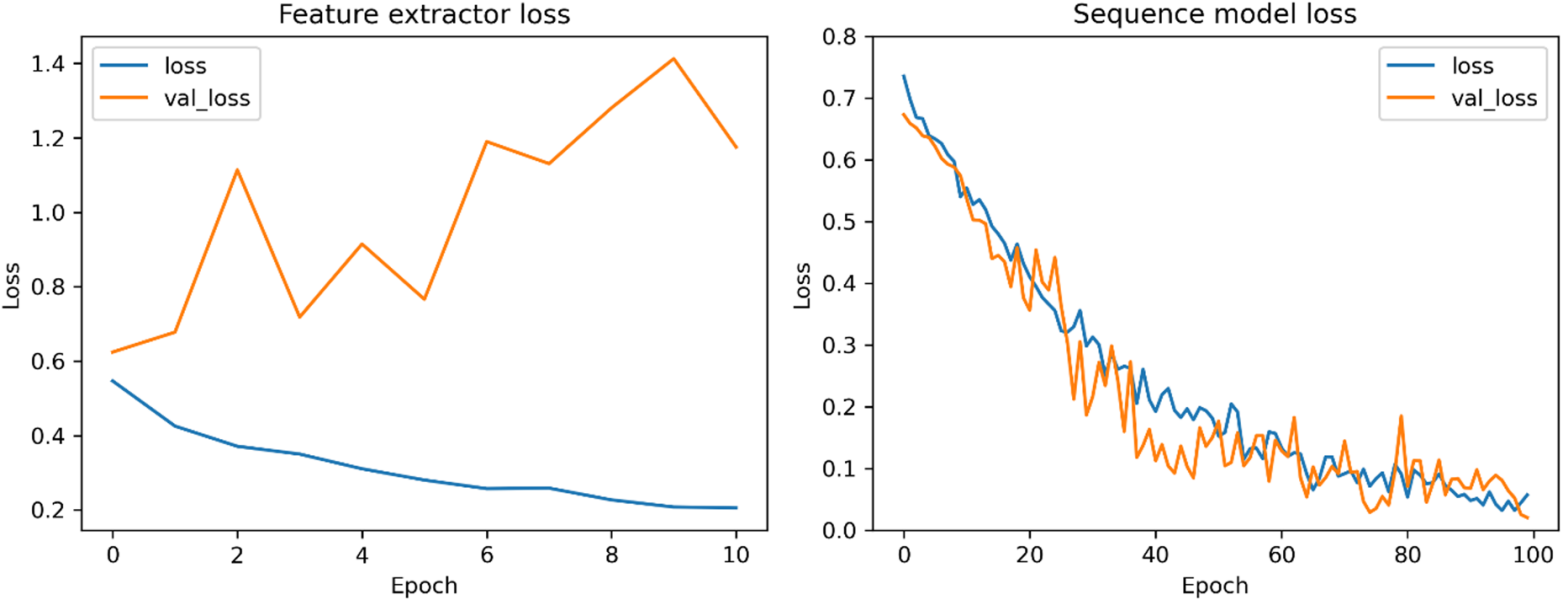
Training and validation loss curves for the feature extractor (CNN) and the full CNN-RNN model (example from fold 7), demonstrating improved convergence and reduced validation loss when incorporating temporal dynamics.

**Fig. 5:**
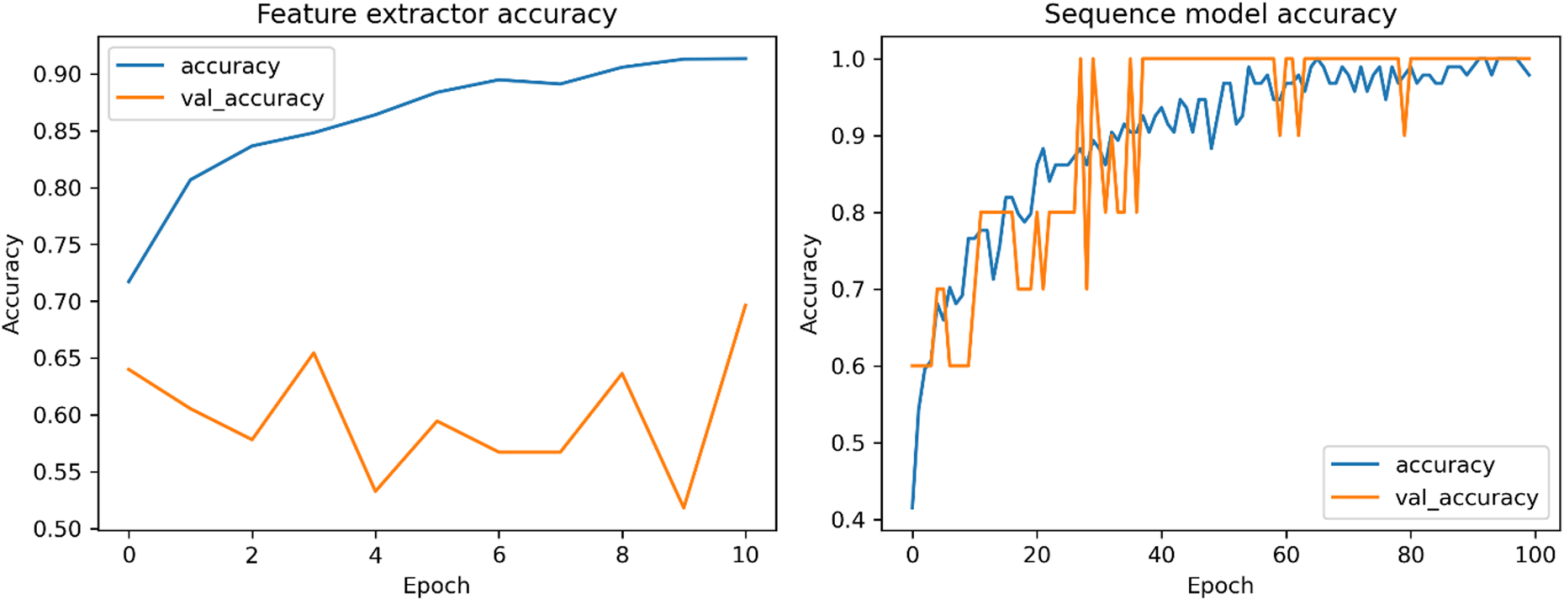
Training and validation accuracy curves for the feature extractor (CNN) and the full CNN-RNN model (example from fold 7), demonstrating improved keratoconus detection capability when incorporating temporal dynamics.

The learning curves for the two parts of the model described above – the feature extractor and the sequential component – are shown in Fig. 4 (example from validation split no. 7). It is evident that the validation loss is higher for the CNN-only model than for the complete model, which confirms that in the diagnosis of keratoconus, analyzing both the corneal topography and its biomechanics yields better results. Additionally, Fig. 5 presents the accuracy values obtained during both training and validation for the two models. It can be observed that relying solely on spatial features results in significantly lower accuracy in keratoconus detection than an approach that incorporates both spatial and temporal features.

**Table 1:** Summarizes the comparison of average values and standard deviations of key performance metrics obtained from 10-fold cross-validation for the model without edge detection and the model with the full preprocessing procedure.

Preprocessing	Accuracy	Precision	Recall	F1-Score	AUC
Without edge detection	0.77 ± 0.19	0.75 ± 0.20	0.73 ± 0.25	0.73 ± 0.23	0.83 ± 0.17
With edge detection	0.91 ± 0.11	0.91 ± 0.12	0.88 ± 0.19	0.88 ± 0.14	0.95 ± 0.07

Table 1. Mean and standard deviation of classification metrics obtained from 10-fold cross-validation for the model without edge detection and the model with full preprocessing procedure.

The model trained on videos with edge detection applied achieved an average accuracy of 0.91 ± 0.11, with similarly high precision (0.91 ± 0.12) and slightly lower recall (0.88 ± 0.19) and F1-score (0.88 ± 0.14). These results indicate that the model maintains consistently strong performance across different data splits. Although the standard deviations reflect moderate variability – particularly in the F1-score and recall – the overall values remain within acceptable bounds. This supports the conclusion that the classifier is not overly sensitive to the particular training/test split and demonstrates good generalization to unseen data. It is also worth noting the significant difference in performance between the model without preprocessing (i.e., without edge detection) and the model employing the full video preparation pipeline. This demonstrates that an appropriate data preprocessing strategy is crucial and can substantially improve the accuracy of keratoconus detection. While Table 1 provides summary statistics, the accompanying boxplots offer a more detailed view of the metric’s distribution. They reveal the presence of outliers, variability between folds, and the symmetry or skewness of the metric values - information that mean and standard deviation alone cannot capture. Notably, such visualizations help identify individual folds where performance may drop significantly, which might otherwise be hidden in the average values. Taken together, the tabular and graphical results offer a more comprehensive assessment of the model’s stability and robustness.

**Fig. 6:**
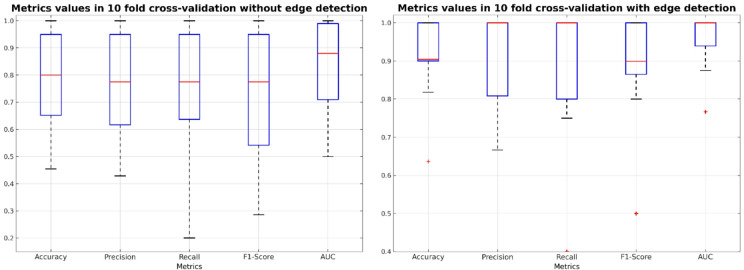
Boxplots of classification performance metrics (Accuracy, Precision, Recall, F1-score, and AUC) across 10-fold cross-validation for the model without edge detection and the model with full preprocessing procedure, illustrating median performance, interquartile range, and presence of outliers.

Figure 6 presents boxplots for Accuracy, Precision, Recall, F1-Score, and Area Under the Receiver Operating Characteristic Curve (AUC) obtained from 10-fold cross-validation for the model without edge detection and the model with full preprocessing procedure. Each boxplot reflects the spread of the metric across the folds: the red line indicates the median, the blue box spans the interquartile range (IQR) - from the first quartile (Q1) to the third quartile (Q3), the whiskers extend to values within 1.5 × IQR, and red plus signs (+) represent outliers.

The boxplots in Fig. 6 illustrate the distributions of core evaluation metrics across all folds for the model without edge detection and for the model with Canny edge detection. For the model with a full preprocessing procedure, all metrics exhibit high median values, ranging from approximately 0.9 to 1.00, suggesting strong and consistent model performance. The box plots show that the median values of precision, recall, and AUC reached 1.0, indicating high performance in keratoconus detection across most validation splits. At the same time, outliers were observed across all metrics, with values ranging from 0.40 to 0.76. Further inspection showed that validation split no. 4 was the most challenging for the model. This may reflect the presence of more ambiguous cases or lower-quality recordings in that subset. The comparison of box plots also indicates that the proposed video preprocessing procedure, including edge detection, improved the model’s stability and robustness. Improvements were observed across all evaluated metrics, with the largest gain in precision and recall, whose median values increased by approximately 0.22.

**Fig. 7:**
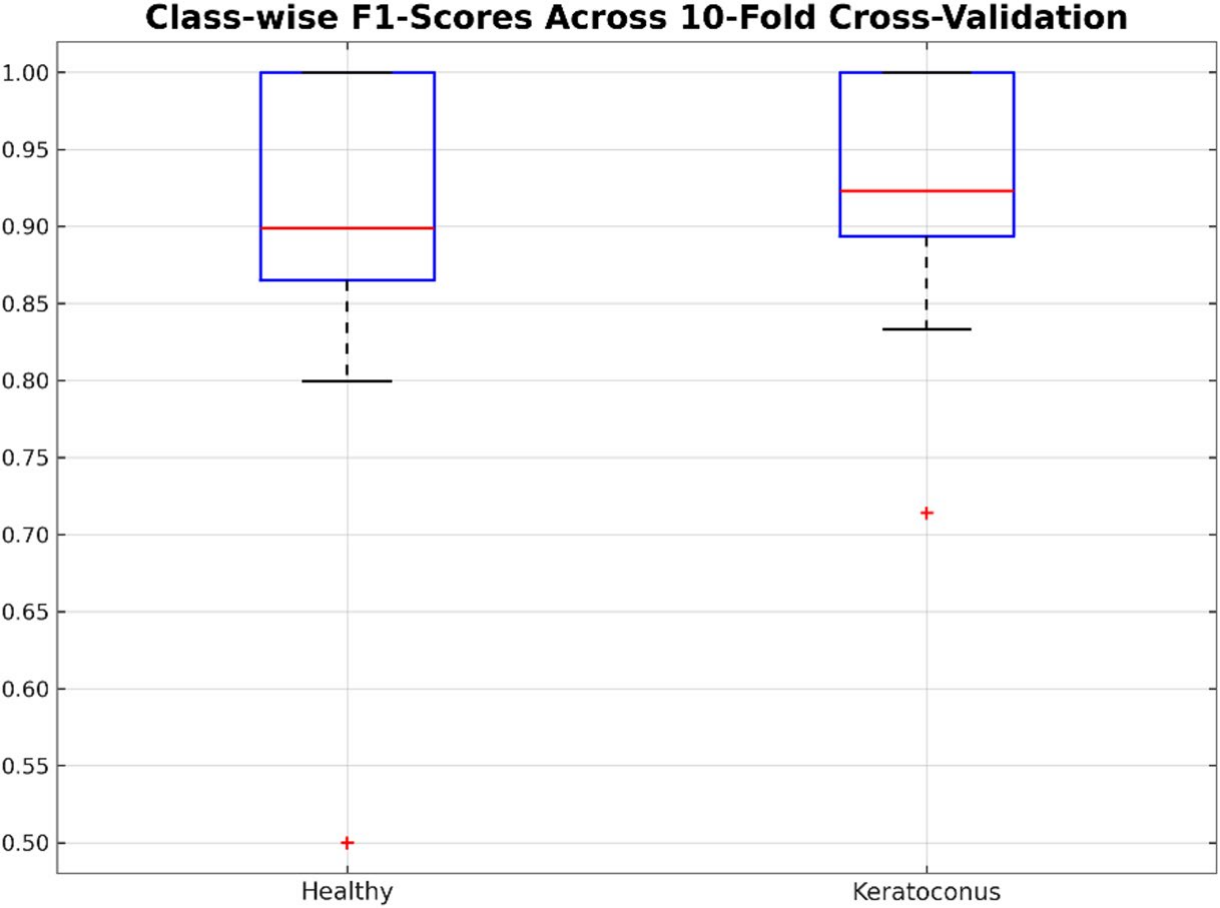
Class-wise distribution of F1-scores for Healthy and Keratoconus cases across 10-fold cross-validation, highlighting higher variability and occasional misclassification in the Healthy class compared to more stable performance in Keratoconus detection.

In the context of medical diagnostics, it is critical to evaluate F1-scores separately for each class, as high average metrics - such as those in Table 1 may mask poor performance on individual classes. Figure 7 addresses this by presenting boxplots of the F1-score for each class (Healthy and Keratoconus) across the 10 folds of cross-validation.

Figure 7 reveals consistent class-wise performance across the folds. Both the Healthy and Keratoconus classes achieve high median F1 scores of approximately 0.90–0.92, suggesting that the model is effective at identifying both conditions. However, subtle differences are evident: the Keratoconus class demonstrates higher consistency, with a narrower interquartile range and fewer extreme values. In contrast, the Healthy class shows greater variability and includes a noticeable outlier near 0.50, suggesting that, in at least one fold, the model had difficulty correctly classifying healthy samples. This may be due to class imbalance or overlapping feature distributions. Despite these deviations, the majority of F1-scores for both classes remain above 0.80, reflecting reliable overall performance and good generalization across classes.

To further evaluate whether the proposed CNN-RNN architecture provides advantages over alternative video-based deep learning approaches, we additionally compared its performance with two baseline models: a 3D CNN^45^ and a Transformer-based architecture^46^. This comparison was performed using the same 10-fold cross-validation protocol and the same evaluation metrics, allowing direct assessment of the contribution of the proposed hybrid spatial-temporal modeling strategy.

**Table 2:** Presents a baseline comparison of the proposed CNN-RNN architecture with two alternative deep learning approaches for video-based keratoconus detection: a 3D CNN model and a Transformer-based ViViT architecture.

Model	Accuracy	Precision	Recall	F1-Score	AUC
CNN-RNN (proposed)	0.91 ± 0.11	0.91 ± 0.12	0.88 ± 0.19	0.88 ± 0.14	0.95 ± 0.07
3D CNN [45]	0.84 ± 0.09	0.85 ± 0.13	0.81 ± 0.14	0.81 ± 0.09	0.85 ± 0.13
Transformer [46]	0.75 ± 0.09	0.75 ± 0.29	0.62 ± 0.33	0.63 ± 0.26	0.74 ± 0.11

Table 2. Mean and standard deviation of classification metrics obtained from 10-fold cross-validation for the compared models.

The proposed CNN-RNN model achieved the highest performance across all evaluated metrics, including Accuracy (0.91 ± 0.11), Precision (0.91 ± 0.12), Recall (0.88 ± 0.19), F1-score (0.88 ± 0.14), and AUC (0.95 ± 0.07). In comparison, the 3D CNN model achieved lower performance, particularly for AUC (0.85 ± 0.13), whereas the Transformer-based model demonstrated the weakest overall classification performance and substantially higher variability across validation folds.

Statistical comparison between models was performed using the Wilcoxon signed-rank test across the 10 cross-validation folds. The proposed CNN-RNN architecture achieved significantly higher AUC values compared with both the 3D CNN baseline (*p* = 0.027) and the Transformer-based model (*p* = 0.004). Additionally, statistically significant improvements over the Transformer model were observed for Accuracy (*p* = 0.016) and F1-score (*p* = 0.012). The remaining metrics also showed consistently higher median values for the proposed architecture, although not all comparisons reached the conventional significance threshold.

**Fig. 8:**
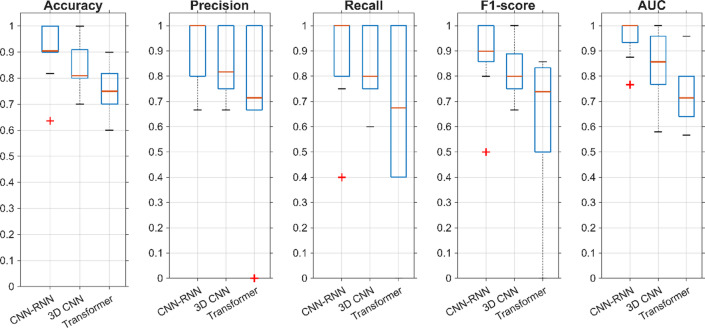
Distribution of classification performance metrics across 10-fold cross-validation for the proposed CNN-RNN model and the compared baseline architectures.

The comparison of metric distributions across validation folds is presented in Fig. 8.

The proposed CNN-RNN model demonstrated not only higher median performance but also lower fold-to-fold variability, indicating improved robustness and generalization. In contrast, the Transformer-based architecture exhibited markedly larger dispersion of results, particularly for Recall and F1-score, suggesting reduced stability under limited-data conditions. These findings indicate that combining convolutional spatial feature extraction with recurrent temporal modeling provides a more reliable representation of corneal deformation dynamics than either volumetric convolution alone or Transformer-based spatiotemporal attention mechanisms.

## Discussion

In recent years, several studies have emerged that use advanced DL architectures for diagnosing keratoconus. Abdelmotaal et al. proposed a hybrid CNN-Transformer model (DenseNet + self-attention) for analyzing dynamic Scheimpflug images, achieving a sensitivity of 93%, specificity of 84%, and an AUC of 0.97^47^. Similarly, Ismael et al. integrated multimodal corneal data (topography, aberrometry, pachymetry, biomechanics) into a Transformer network, which significantly outperformed traditional SVM, RF, and classical CNN models in terms of accuracy and other metrics^48^. Our accuracy of ~ 0.90 is within the same range, suggesting that Transformer-like architectures can further improve recognition performance, indicating that the proposed approach remains competitive. It is worth noting that existing CNN or hybrid CNN+LSTM models typically achieved comparable results; however, they also suffer from a “black box” nature. A growing number of ophthalmology studies employ explainability techniques – e.g., Grad-CAM – to highlight image regions relevant to model decisions^49^. Although the proposed model has limited interpretability in the strict deep-learning sense, the input data were strongly constrained through preprocessing and corneal contour extraction, thereby reducing the influence of irrelevant background information and focusing the analysis on clinically meaningful deformation patterns.

This study evaluated a deep learning classification model for its ability to distinguish between healthy eyes and those affected by keratoconus using a 10-fold stratified cross-validation framework. The model consistently demonstrated strong performance across all key evaluation metrics, with average values for accuracy, precision, recall, and F1-score hovering around 0.90 and an AUC of about 0.95. These results indicate that the model is effective within the available cohort and shows promising internal generalization across varying training and validation splits.

The application of 3D CNN models for keratoconus detection based on the collected dataset was investigated in another study^45^, in which the best-performing model achieved an average accuracy of approximately 89%. The present study proposes a model that slightly improves the performance of keratoconus detection. Moreover, it employs transfer learning by fine-tuning only the last 20 layers of the InceptionV3 model, enabling a significant reduction in training time. Additional comparative experiments in the present study demonstrated that the proposed CNN-RNN architecture also outperformed both 3D CNN and Transformer-based baseline models in overall classification stability and AUC, supporting the effectiveness of combined spatial-temporal feature modeling for dynamic corneal deformation analysis.

Low variability in accuracy and AUC further supports the model’s robustness. The model achieves very strong performance for the vast majority of validation splits, as evidenced by the high median values of precision, recall, and AUC. Further analysis confirmed that one of the folds was particularly challenging, likely because of a higher proportion of outlier samples. The results also indicate that incorporating edge detection during data preprocessing significantly improves the model’s stability.

Class-wise evaluation revealed subtle differences in model behavior. Although both the Healthy and Keratoconus classes achieved high median F1-scores, the Healthy class exhibited greater variability, including a single outlier. This suggests that the model may be less stable in some healthy cases, potentially due to feature overlap or class imbalance. Such behavior could have practical implications in clinical settings, such as an increased risk of false-positive diagnoses, particularly in screening-oriented settings, where high sensitivity must be balanced with an acceptable false-positive rate.

These findings emphasize the importance of complementing global metrics with per-class evaluations when assessing machine learning models for medical diagnostics. While averaged results can mask fold-specific or class-specific shortcomings, visual tools such as boxplots provide a richer, more nuanced understanding of model behavior. Overall, the proposed model presents a strong and promissing foundation for keratoconus detection, though further validation on external, diverse datasets is necessary.

An important issue is the limitations of the data sample used. Our analysis is based on a relatively small, single-center set of 104 measurements, which may limit the generalizability of the results. Recent works highlight that most DL models in keratoconus have been trained on small cohorts, often without external validation^50^.

It should be emphasized that the present study has a pilot character, reflecting both the exploratory nature of the proposed deep learning framework and the limited availability of high-quality corneal deformation recordings. In routine clinical practice, acquiring large and well-balanced datasets of dynamic CORVIS recordings remains difficult. In addition, the proposed pipeline was specifically designed for sequential image data extracted from CORVIS recordings, which limits the immediate availability of compatible external datasets acquired under comparable imaging protocols. Therefore, the dataset size should be interpreted in the context of these practical constraints rather than as a methodological shortcoming alone. While the use of stratified 10-fold cross-validation at the patient level reduces the risk of overfitting and data leakage, it does not replace true external validation, and the obtained performance estimates may still be subject to variability and optimistic bias. Therefore, further validation on independent, external datasets and multi-center cohorts is necessary to confirm the generalizability and robustness of the proposed model.

## Limitations and Future Plans

Despite the promising outcomes, several limitations of this study must be acknowledged:

1. **Dataset size and diversity**: The model was evaluated using 10-fold cross-validation on a relatively limited dataset. Although this method helps reduce overfitting and assess generalization, the dataset may not reflect the full spectrum of clinical variability – such as different stages of keratoconus, comorbidities, or image acquisition inconsistencies. The present study should therefore be regarded as a pilot/proof-of-concept investigation, particularly limited by the availability of carefully curated dynamic CORVIS recordings.
2. **Class imbalance and sensitivity**: While overall performance was high, class-wise analysis revealed greater variability and an outlier for the Healthy class. This may indicate underlying class imbalance or less distinct feature representations in healthy eyes, increasing the potential for false positives in practice.
3. **Lack of external validation**: The model’s performance has not been validated on independent datasets or across multiple institutions. Its ability to generalize to external populations remains unproven. Although patient-level splitting was applied to prevent data leakage and strengthen internal evaluation, this does not substitute for validation on fully independent cohorts.
4. **Limited interpretability**: As with many deep learning models, particularly black-box classifiers, the current approach lacks clinical interpretability. At the same time, this limitation is partly mitigated by the adopted preprocessing strategy and corneal contour extraction, which constrain the input representation to deformation patterns of clinical relevance.

The results obtained in this study indicate the high effectiveness of the proposed model, but they also reveal important directions for further analysis. In particular, the observed greater variability in the results for the healthy class and the presence of individual outliers (Recall ~ 0.40) suggest the need for a more in-depth analysis of the model’s decision boundary and the characterization of common features for difficult-to-classify cases. Future research should utilize feature space exploration methods (e.g., embeddings) and error analysis, which may help identify subtle biomechanical patterns leading to misclassification. Expanding the dataset to include borderline cases (subclinical forms of keratoconus) could further enhance the model’s ability to detect early pathological changes^12,51,52^.

Another important direction of development is validation of the model on external, multi-center datasets, which will allow assessment of its true generalization ability in clinical settings. Current limitations related to the relatively small sample size and its homogeneity indicate the need to integrate data from different populations and diagnostic devices. In this context, a multimodal approach, combining biomechanical data with topographic and tomographic information, and clinical parameters, seems particularly promising. This could lead to further improvements in diagnostic performance and increased model robustness to input data variability.

An important element of future research should also be increasing the interpretability of the model through the implementation of explainable AI methods (e.g., Grad-CAM), enabling the identification of image regions and phases of corneal deformation crucial for decision-making. This will not only increase clinical confidence but also provide a better understanding of the biomechanical mechanisms involved in the development of keratoconus. Furthermore, the development of architectures based on transformers or hybrid CNN-Transformer approaches, as well as the optimization of key timeframe selection, could further improve model performance. In the long term, it is also worthwhile to develop real-time clinical decision support systems integrated directly with diagnostic devices.

## Conclusions

This study demonstrates that the proposed deep learning model achieves high and consistent performance in classifying healthy versus keratoconic eyes. Across 10-fold cross-validation, the model maintained average accuracy, precision, recall, and F1-score around 0.90, indicating strong generalization and stability. Class-wise analysis confirmed the model’s effectiveness in detecting both conditions, though slightly greater variability was noted for the Healthy class.

By combining aggregate and class-specific evaluation metrics with boxplot visualizations, the study provides a comprehensive view of model reliability and performance variation across data splits. These findings support the model’s potential as a reliable tool for early keratoconus detection or screening. Its performance suggests that it could effectively complement current diagnostic workflows, potentially reducing misdiagnosis and enhancing clinical decision-making. Nevertheless, additional testing on independent and diverse datasets is required before clinical integration.

## Data Availability

The datasets generated and/or analyzed during the current study are not publicly available due to patient privacy and ethical restrictions but are available from the corresponding author on reasonable request.

## Code availability

The complete Python pipeline used for video preprocessing, model training, evaluation, and figure generation is publicly available in Zenodo at 10.5281/zenodo.19312928.
